# Rapid intraoperative diagnosis of pediatric brain tumors using Raman spectroscopy: A machine learning approach

**DOI:** 10.1093/noajnl/vdac118

**Published:** 2022-07-26

**Authors:** Rashad Jabarkheel, Chi-Sing Ho, Adrian J Rodrigues, Michael C Jin, Jonathon J Parker, Kobina Mensah-Brown, Derek Yecies, Gerald A Grant

**Affiliations:** Department of Neurosurgery, Stanford University, Stanford, California, USA; Department of Neurosurgery, University of Pennsylvania, Philadelphia, Pennsylvania, USA; Department of Applied Physics, Stanford University, Stanford, California, USA; Department of Neurosurgery, Stanford University, Stanford, California, USA; Department of Neurosurgery, Stanford University, Stanford, California, USA; Department of Neurosurgery, Stanford University, Stanford, California, USA; Department of Neurosurgery, University of Pennsylvania, Philadelphia, Pennsylvania, USA; Department of Neurosurgery, Stanford University, Stanford, California, USA; Department of Neurosurgery, Stanford University, Stanford, California, USA; Department of Neurosurgery, Duke University, Durham, North Carolina, USA

**Keywords:** machine learning, pediatric brain tumors, Raman spectroscopy

## Abstract

**Background:**

Surgical resection is a mainstay in the treatment of pediatric brain tumors to achieve tissue diagnosis and tumor debulking. While maximal safe resection of tumors is desired, it can be challenging to differentiate normal brain from neoplastic tissue using only microscopic visualization, intraoperative navigation, and tactile feedback. Here, we investigate the potential for Raman spectroscopy (RS) to accurately diagnose pediatric brain tumors intraoperatively.

**Methods:**

Using a rapid acquisition RS device, we intraoperatively imaged fresh ex vivo brain tissue samples from 29 pediatric patients at the Lucile Packard Children’s Hospital between October 2018 and March 2020 in a prospective fashion. Small tissue samples measuring 2-4 mm per dimension were obtained with each individual tissue sample undergoing multiple unique Raman spectra acquisitions. All tissue samples from which Raman spectra were acquired underwent individual histopathology review. A labeled dataset of 678 unique Raman spectra gathered from 160 samples was then used to develop a machine learning model capable of (1) differentiating normal brain from tumor tissue and (2) normal brain from low-grade glioma (LGG) tissue.

**Results:**

Trained logistic regression model classifiers were developed using our labeled dataset. Model performance was evaluated using leave-one-patient-out cross-validation. The area under the curve (AUC) of the receiver-operating characteristic (ROC) curve for our tumor vs normal brain model was 0.94. The AUC of the ROC curve for LGG vs normal brain was 0.91.

**Conclusions:**

Our work suggests that RS can be used to develop a machine learning-based classifier to differentiate tumor vs non-tumor tissue during resection of pediatric brain tumors.

**Key Points**

- Raman spectroscopy can rapidly differentiate normal brain from tumor tissue.
- Raman spectroscopy can rapidly differentiate normal brain from low-grade glioma tissue.

**Importance of the Study**

Here, we show with standard machine learning techniques that Raman spectroscopy can be used to rapidly classify pediatric brain tumors. To the best of our knowledge, we have curated the largest dataset of Raman spectra generated from fresh pediatric brain tissue labeled with their corresponding final histopathology diagnosis. The accuracy of our tissue classifiers which can distinguish normal brain from tumor tissue (89.8%), and the more difficult task of normal brain from low-grade glioma tissue (86.2%), has the potential to improve the extent of surgical resection, which for many pediatric brain tumor subtypes is associated with improved survival. Through future studies with larger datasets, across multiple institutions, we seek to apply Raman spectroscopy to not only distinguish normal brain from any tumor or LGGs, but more specifically classify tumors by their type, WHO grade, and molecular subclassification.

Pediatric brain tumors are the most common solid tumors in children and the leading cause of cancer deaths in children aged 0-14 years old.^1^ Surgical resection is a mainstay of treatment in these patients both for tissue diagnosis and tumor debulking. Greater extent of surgical resection has been shown to improve progression-free survival and overall survival across many pediatric brain tumor subtypes.^2–5^ While maximal resection of tumor is desired, neurosurgeons are limited by the challenge of differentiating normal brain from tumor, in particular for low-grade tumors. Currently, intraoperative microscopic visual inspection, intraoperative navigation, intraoperative MR imaging (iMRI), and tactile feedback while manipulating tissue comprise the primary methods to differentiate tumor margins. The gold standard of intraoperative analysis of a “frozen” tissue sample by a pathologist is limited by the lengthy iteration time between samples, making it impractical for minute-to-minute surgical decision making. Furthermore, iMRI is resource intensive, only available in select centers, and is often technically cumbersome making obtaining more than one scan during a case a challenge. In response to this challenge, various advanced tissue imaging techniques have been investigated to enhance a surgeon’s ability to detect tumor, including intraoperative ultrasonography, fluorescence microscopy, and more recently Raman spectroscopy (RS).^6–13^ Here, we sought to investigate whether RS can be used to rapidly diagnose pediatric brain tumor samples.

RS is an emerging, rapid, non-destructive imaging technique based on the Raman effect. In brief, when light is incident on a molecule it can either be absorbed or scattered. There are two types of scattering: Rayleigh scattering and Raman scattering. The majority of scattered light is from Rayleigh scattering, which is light that is scattered back at the same energy as the incident light. Raman scattering makes up a small fraction of scattered light and describes light that is scattered with a different energy from the incident light due to an exchange of energy with a molecule’s bonds. In Raman scattering, the emitted light has a different energy, and thus a different frequency from incident light and this emitted light can be captured by a spectrophotometer. RS takes advantage of Raman scattering for material identification as the different molecular compositions of a given tissue produce a unique Raman spectra or signature.^14–18^ There has been significant interest focused on the material identification properties of RS for enhancing detection of brain tumor margins intraoperatively. RS has been used to differentiate white matter from gray matter, tumor from surrounding necrosis, and even tumor from normal brain.^11,19–21^ Unfortunately, the bulk of RS studies to date have either been performed in the adult context, limited to pediatric frozen brain or formalin-fixed brain sections, or in animal models.^22,23^ In this work, we explored the potential of RS to detect pediatric brain tumor margins by creating a machine learning model using a dataset of pediatric brain tumor tissue imaged with RS intraoperatively at the time of surgery.

## Methods

### Study Cohort

This study was approved by the Stanford University Institutional Review Board panel on Medical Human Subjects (IRB #43701). Written informed consent was obtained from the parent or legal guardian of all pediatric patients included in the study. All patients included in the study underwent brain tissue resection as part of tumor debulking or epilepsy surgery (normal controls) by our corresponding author (G.A.G.) between October 2018 and March 2020 at the Lucile Packard Children’s Hospital (Palo Alto, CA, USA) in a prospective fashion.

### Study Design

**Figure 1.**
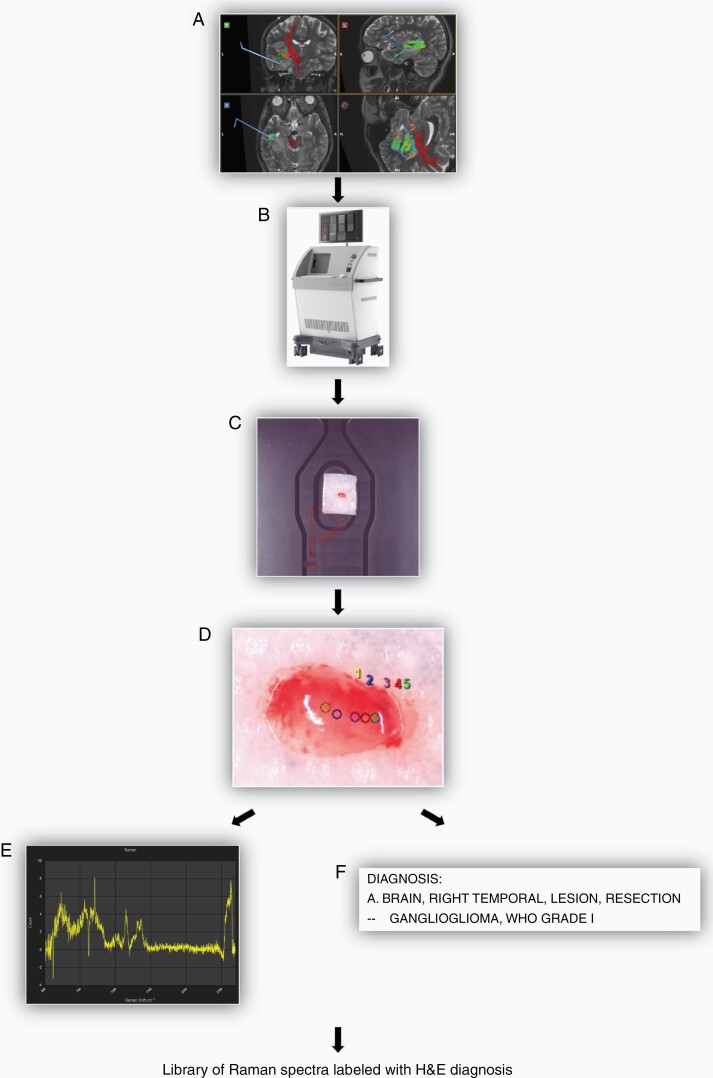
Overview of intraoperative, ex vivo, Raman spectra acquisition workflow. (A) Tissue resected using frameless stereotaxy. (B) Tissue sample placed in Solais device located in operating room. (C) Sample size limited to 2-4 mm per dimension (length × width × height) and placed on saline moistened telfa inside of petri dish. (D) Raman points selected for spectra acquisition. (E) Example of Raman spectra generated in seconds. (F) All tissue samples sent for individual histopathology review after Raman spectra acquisition.

Using a rapid acquisition RS device (Solais, Synaptive Medical, Toronto, ON, Canada) we intraoperatively imaged fresh ex vivo pediatric brain tissue (Figure 1). Core tumor or normal brain regions were localized using frameless stereotaxy. As tissue was resected it was immediately placed on saline moistened telfa inside of a petri dish and then placed in the Solais device for imaging (Figure 1A and B). Tissue samples were limited in size to 2-4 mm per dimension to ensure maximal spatial correlation between acquired Raman spectra and final histopathologic diagnosis (Figure 1C). In cases where a resected tissue sample was larger than 2-4 mm per dimension, it was divided in the field by the surgeon prior to imaging. Once a sample was placed in the Solais, using the device’s built-in camera to zoom, points were selected for Raman spectra acquisition. Raman points were selected in the center of the tissue sample and in avoidance of areas contaminated with blood products. Each tissue sample had between 1 and 5 Raman points selected for spectra acquisition, with approximately 5 seconds required for spectra acquisition. The short acquisition time allowed for high throughput analysis of multiple samples per patient (Figure 1D and E). After tissue had been imaged, individual pieces were separately labeled and catalogued, placed in formalin, and transported to the Stanford Hospital neuropathology laboratories for final histopathological diagnosis as RS is a label-free, non-destructive imaging technique (Figure 1F). Using the above-described approach, a large labeled library of brain tissue samples, which were analyzed with RS, and which also underwent formal histopathological review, was created for the development of a tissue classifier.

### Raman Spectroscopy

Raman spectra were acquired using the Solais (Synaptive Medical) (Figure 1B). The Solais is a multi-purpose, research device capable of visible light imaging, RS, and polarization-sensitive optical coherence tomography. The Lucile Packard Children’s Hospital is the only children’s hospital in the United States to have used this device. The Solais’ Raman microscope has an excitation wavelength of 785 nm, excitation power of 50 mW, bandpass of 200-3000 cm^−1^, and spectral resolution of 6 cm^−1^. All Raman spectra were obtained using an averaging number of 5 and an accumulation time of 1 second. Each Raman point has an approximate depth penetration of 1 mm and a field-of-view of 200 microns. Accuracy and calibration of the Solais device were assessed with spectra acquisition on a silicon standard prior to each case.

### Model Development

#### Data preprocessing.

—Spectra were individually normalized by the maximum intensity value. The model pipeline consisted of principal component analysis (PCA) for dimensionality reduction from 1614 features to 100 features, followed by L2-normalized logistic regression trained using the limited-memory Broyden-Fletcher-Goldfarb-Shanno optimization algorithm. The number of output dimensions for PCA was chosen so that >95% of the variance in the training dataset would be represented.

#### Model evaluation.

—Performance metrics were calculated using leave-one-patient-out cross-validation (LOPOCV), where all samples and spectra associated with each patient were held out from the training set at a time. The model was tested on all spectra and samples from this held-out patient, and then the process was repeated for all patients included in each classification task. Predictions for all patients were then aggregated to calculate performance metrics. Each prediction is a probabilistic value and is thresholded using Youden’s J metric to report accuracy, sensitivity, and specificity.

## Results

### Patients

A total of 29 pediatric patients were included in this study yielding 160 unique tissue samples and 678 unique Raman spectra (Table 1). Tumor tissue was obtained from 20 patients. Normal tissue was obtained from 12 patients. In specific cases, normal tissue samples were obtained from patients from which tumor tissue samples were also obtained. A total of 11 unique tumor diagnoses were obtained from the 20 patients with tumors, 8 of whom had low-grade gliomas (LGGs) (Table 2). LGGs included ganglioglioma, angiocentric glioma, and pilocytic astrocytoma. Samples with indeterminate final pathology were excluded from our labeled library. Specifically, we excluded a single patient who underwent resection of what radiographically was favored to be an LGG, but whose final pathology returned inconclusive for all 15 submitted tissue samples.

**Table 1.** Number of Patients, Samples, and Spectra per Classification Task

Classification	Number of Patients	Number of Samples	Number of Spectra
Total	29	160	678
Tumor vs Normal			
Tumor	20	105	459
Normal	12	55	219
LGG vs Normal			
LGG	8	44	196
Normal	12	55	219

Abbreviation: LGG, low-grade glioma.

**Table 2.** Number of Patients, Samples, and Spectra per Tumor Subtype

Tumor Pathologies	Number of Patients	Number of Samples	Number of Spectra
Pilocytic astrocytoma	4	22	93
Ependymoma	4	15	64
Ganglioglioma	3	18	85
Medulloblastoma	1	8	26
Glioblastoma	1	7	34
Teratoma	1	2	9
ATRT	1	6	27
Choroid plexus papilloma	2	15	68
Embryonal tumor	1	7	32
Craniopharyngioma	1	1	3
Angiocentric glioma	1	4	18

Abbreviation: ATRT, Atypical Teratoid Rhabdoid Tumor.

### Tumor vs Normal Brain

**Figure 2.**
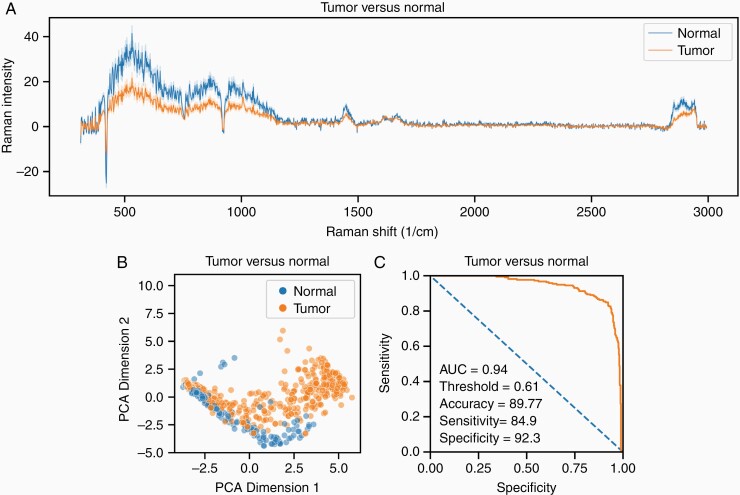
Tumor vs normal. (A) Representative tumor vs normal brain spectra with associated 95% confidence interval variance bands. (B) PCA-based two-dimensional sorting of tumor spectra from normal brain spectra. (C) ROC curve of a trained logistic regression model tasked with classifying tissue samples as either tumor or normal brain using LOPOCV. Abbreviations: LOPOCV, leave-one-patient-out cross-validation; PCA, principal component analysis, ROC, receiver-operating characteristic curve.

A total of 20 patients yielding 105 tissue samples and 459 Raman spectra had a final pathology classification as neoplastic tissue. A total of 12 patients yielding 55 tissue samples and 219 Raman spectra had a final pathology classification as normal brain tissue. Figure 2A shows a representative tumor vs normal brain spectra with associated 95% confidence interval variance bands. Figure 2B shows a PCA-based two-dimensional sorting of tumor spectra from normal brain spectra. Figure 2C shows the receiver-operating characteristic (ROC) curve of a trained logistic regression model tasked with classifying tissue samples as either tumor or normal brain. The classifier achieved an area under the curve (AUC) of 0.94 with an accuracy of 89.8%, sensitivity of 84.9%, and specificity of 92.3%.

### LGG vs Normal Brain

**Figure 3.**
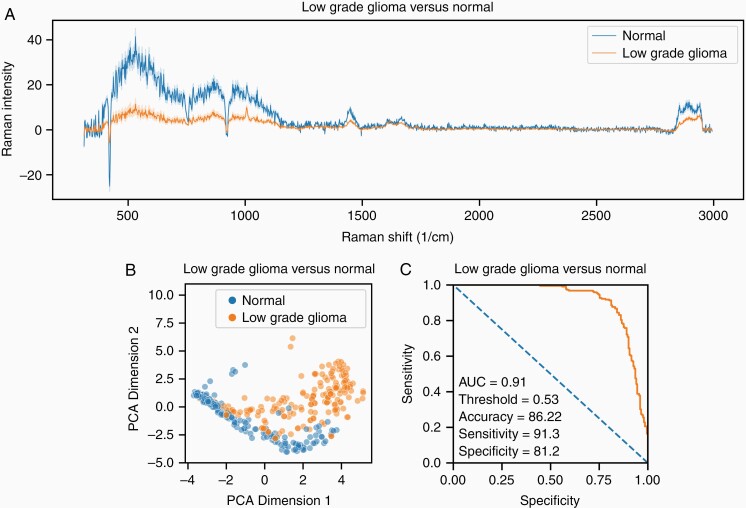
Low-grade glioma vs normal. (A) Representative low-grade glioma vs normal brain spectra with associated 95% confidence interval variance bands. (B) PCA-based two-dimensional sorting of low-grade glioma spectra from normal brain spectra. (C) ROC curve of a trained logistic regression model tasked with classifying tissue samples as either tumor or normal brain using LOPOCV. Abbreviations: LOPOCV, leave-one-patient-out cross-validation; PCA, principal component analysis, ROC, receiver-operating characteristic curve.

A total of 8 patients yielding 44 tissue samples and 196 Raman spectra had a final pathology classification as LGG. The same normal brain dataset consisting of 12 patients yielding 55 tissue samples and 219 Raman spectra that were used to create the tumor vs normal brain classifier was used to develop the LGG vs normal brain classifier. Figure 3A shows a representative LGG vs normal brain spectra with associated 95% confidence interval variance bands. Figure 3B shows a PCA sorting LGG spectra from normal brain spectra. Figure 3C shows the ROC curve of a trained logistic regression model tasked with classifying tissue samples as either LGG or normal brain. The classifier achieved an AUC of 0.91 with an accuracy of 86.2%, sensitivity of 91.3%, and specificity of 81.2%.

## Discussion

Here, we show that with standard machine learning techniques, the material identification properties of RS can be harnessed for rapid classification of pediatric brain tumors. To the best of our knowledge, we have curated the largest dataset of Raman spectra generated from fresh pediatric brain tissue labeled with their corresponding final histopathology diagnosis. The accuracy of our tissue classifiers distinguishing normal brain from tumor (89.8%), and the more difficult task of normal brain from LGGs (86.2%), has translational potential for clinical impact as the extent of resection for most pediatric brain tumors, including LGGs and ependymomas, is associated with improved progression-free survival and overall survival.^5,24,25^ Our classifier discriminating normal brain from LGGs is of particular importance as (1) LGGs are the most common pediatric brain tumors accounting for ~30% of all pediatric brain tumors and (2) by nature of being WHO grade I, LGGs are the most similar to normal brain, and thus the most difficult to distinguish visually intraoperatively.^26^ For example, a recent multicenter study showed that 43.6% of pediatric LGG patients required additional surgery after a single iMRI scan.^27^

While our study represents the largest labeled dataset of fresh pediatric brain tissue currently published, it is still limited by a relatively small sample size in the context of the large datasets which are more optimal for training machine learning models. Through future studies with larger datasets across other institutions, we seek to apply RS to not only distinguish normal brain from any tumor or LGGs, but more specifically classify tumors by their type, WHO grade, and possibly molecular subgroup. We envision that future work by our group and others will develop RS into a fully developed and easily deployed tool in the neurosurgeon’s armamentarium.
